# Comparison of Quantitative Mass Spectrometric Methods for Drug Target Identification by Thermal Proteome Profiling

**DOI:** 10.1021/acs.jproteome.3c00111

**Published:** 2023-07-13

**Authors:** Amy L. George, Frances R. Sidgwick, Jessica E. Watt, Mathew P. Martin, Matthias Trost, José Luis Marín-Rubio, Maria Emilia Dueñas

**Affiliations:** † Laboratory for Biological Mass Spectrometry, Biosciences Institute, Newcastle University, Newcastle-upon-Tyne NE2 4HH, U.K.; ‡ Newcastle Cancer Centre, Northern Institute for Cancer Research, Medical School, Newcastle University, Paul O’Gorman Building, Framlington Place, Newcastle upon Tyne NE2 4HH, U.K.

**Keywords:** thermal proteome profiling (TPP), data-independent acquisition (DIA), tandem mass tag (TMT), data-dependent acquisition (DDA), spectral library, hybrid library, target deconvolution, acute myeloid leukemia (AML)

## Abstract

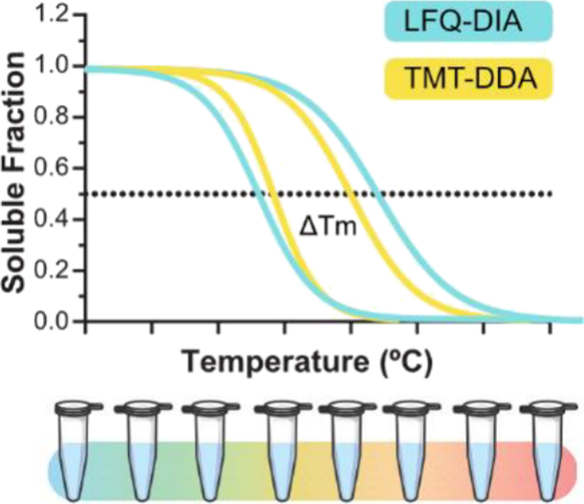

Thermal proteome profiling (TPP) provides a powerful approach to studying proteome-wide interactions of small therapeutic molecules and their target and off-target proteins, complementing phenotypic-based drug screens. Detecting differences in thermal stability due to target engagement requires high quantitative accuracy and consistent detection. Isobaric tandem mass tags (TMTs) are used to multiplex samples and increase quantification precision in TPP analysis by data-dependent acquisition (DDA). However, advances in data-independent acquisition (DIA) can provide higher sensitivity and protein coverage with reduced costs and sample preparation steps. Herein, we explored the performance of different DIA-based label-free quantification approaches compared to TMT-DDA for thermal shift quantitation. Acute myeloid leukemia cells were treated with losmapimod, a known inhibitor of MAPK14 (p38α). Label-free DIA approaches, and particularly the library-free mode in DIA-NN, were comparable of TMT-DDA in their ability to detect target engagement of losmapimod with MAPK14 and one of its downstream targets, MAPKAPK3. Using DIA for thermal shift quantitation is a cost-effective alternative to labeled quantitation in the TPP pipeline.

## Introduction

Target deconvolution is a key step in the drug discovery pipeline for validating compound-target engagement, determining the mechanism of action and probing interactions with unexpected proteins to identify possible new therapeutic targets and off-target toxicities.^1^ Thermal proteome profiling (TPP) provides a powerful approach for studying proteome-wide interactions of therapeutic molecules and their target or off-target proteins.^2^ It combines the principles of the cellular thermal shift assay with multiplexed quantitative mass spectrometry (MS), as direct drug binding can elicit conformational changes in a protein, affecting its thermal stability.^3,4^ Cell extracts, which lack normal cellular metabolism, are used to investigate thermal shifts due to direct compound engagement, although proteins are removed from their native environment, which may also affect thermal stability.^2,5,6^ Treatment of intact cells or tissues allows for additional understanding of cellular response in a physiologically relevant setting, affecting protein thermal stability while capturing downstream effects.^2^ The method has been applied to interrogate drug–target interaction, protein–substrate interaction, protein degradation, and post-translational modifications (PTMs) in various biological systems.^6−10^

Quantifying differences in thermal stability requires high quantitative accuracy and consistent detection. Isobaric labeling with tandem mass tags (TMTs) has been traditionally used with data-dependent acquisition (DDA) to quantify proteome-wide changes in thermal stability as the pooling of samples increases throughput, reduces technical variability, and is suitable for fractionation, thus providing deep proteomic coverage.^11^ Initially, 10 temperatures labeled with TMT-10plex^12^ were recommended to generate melting curve data points, requiring two TMT experiments per biological replicate, for treatment and vehicle control conditions.^4^ Since the development of TMTpro 16-plex and 18-plex, performing eight (or nine) temperatures for melting curve generation allows two conditions to be analyzed within a single experiment per biological replicate.^13−15^ Sample-specific reporter-ions produced after fragmentation are used for quantification, reducing the number of missing peptide quantification values as peptides across conditions are not separately subject to semi-stochastic precursor sampling as with label-free DDA quantification but are acquired within the same MS^2^ scan.^16^ While this quantitative approach works very well within the boundaries of its multiplicity, principal drawbacks include the high price of reagents, extended sample preparation time, and constraints on sample number analyses compared with label-free approaches. In addition, notable increases in missing protein and peptide values are observed once multiple batches are integrated.^17^ Increased variability is particularly seen with the cell-based TPP protocol due to sample-specific lysis, requiring additional replicates for reproducible thermal stability shifts to become significant.^4^ High workflow costs frequently restrict data collection, and therefore many TPP studies were performed with just two biological replicates, which can limit result significance.^5,18,19^ Moreover, quantification of MS^2^ fragments can suffer from ion interference and ratio compression, leading to a dampening of fold changes and under-measure of thermal shifts.^20,21^ Synchronous precursor selection (SPS) MS^3^ methods (partly) overcome this by isolating fragments after MS^2^ for further fragmentation, resulting in improved quantitative accuracy, but reduced proteome coverage due to lower scan rates and is limited to Tribrid instruments.^22^

Label-free quantification (LFQ) acquired in DDA has been used as a less expensive alternative to isobaric methods for thermal shift quantitation for target deconvolution. In the work by Türkowsky et al., the gain in flexibility with LFQ-DDA enabled adaptation of the TPP protocol to investigate oxygen-sensitive proteins in anaerobic bacteria. However, low proteomic sequence coverage and a high proportion of missing values in melting curves were reported, likely due to the intensity-triggered precursor selection biases accompanying DDA quantification.^23,24^ Alternatively, data-independent acquisition (DIA) is a single-shot method that provides precise, accurate proteome quantification of label-free samples with low missing values and high analytical depth, as all detectable peptides within specific *m/z* windows are fragmented and quantified in parallel. This quantitative approach has previously been adopted for target deconvolution in limited proteolysis workflows to measure stability of the protein–ligand complex under proteolytic conditions.^25^ More recently, it was adopted in a matrix thermal shift assay to detect ligand concentration-dependent stabilization of proteins, at a single melting temperature.^26^ Ruan et al. treated K562 lysates with staurosporine, a model widely used for evaluation of the thermal shift assay and demonstrated reduction in sample preparation time, cost, and effort. By adopting a library-free, DirectDIA approach, they increased throughput and achieved comparable sensitivity for identifying kinase targets to a recent 2D-TPP TMT study.^26^

There are now various pipelines for DIA peptide identification, each with distinct strengths and suitability depending on the type of proteomic data.^27−29^ Project-specific libraries generated by analyzing pre-fractionated DDA samples, which are representative of the model system, historically provide the greatest proteome coverage but largely depend on the size and quality of the library, increasing instrument run time.^27,30,31^ Hybrid libraries can be constructed to increase library depth and precision by combining the DDA library with DIA data.^32^ However, library-free approaches require no additional data to generate spectral libraries and have been demonstrated to achieve nearly equivalent whole-proteome coverage.^33,34^ Library-free analysis is commonly performed in Spectronaut using DirectDIA, where Pulsar performs a classical database search directly on the DIA runs to create a library, which is then used for a targeted analysis of the same DIA runs.^35^ An alternative approach is to produce an in silico spectral library using software such as DIA-NN or Fragpipe with MS-Fragger-DIA,^36^ which has not yet been trialed for analyzing TPP data.^37^

In this study, we explored the performance of different library-free and library-based DIA approaches in a TPP workflow and benchmarked these with traditional TMT-DDA for thermal shift quantitation. We use losmapimod, a potent MAPK14 ATP competitive inhibitor, to provide a known true positive hit in acute myeloid leukemia (AML) cells. While all DIA workflows reliably measured thermal stabilization of MAPK14 and its downstream effector, MAPKAPK3, library-free mode DIA-NN performed best as an alternative to TMT-DDA for thermal proteome profiling.

## Experimental Section

### Compounds

Losmapimod (GW856553) was purchased from Biorbyt (UK) and human recombinant IL-1β and TNF-α from PeproTech (UK). A clinicaltrials.gov search was conducted on February 13, 2023, for losmapimod (keywords: losmapimod, GW856553, GW856553X, SB856553, or GSK-AHAB).

### Cell Culture

A THP-1 (ATCC, TIB-202) cell line was cultured in RPMI 1640 (Gibco) supplemented with 10% heat-inactivated fetal bovine serum and 2 mM l-glutamine at 37 °C in a humidified 5% CO_2_ atmosphere. ATCC routinely performs cell line authentication, using short tandem repeat profiling as a procedure. Cell experimentation was always performed within a period not exceeding 6 months after resuscitation in mycoplasma-free culture conditions.

### Cell Proliferation and Viability Assay

Cells were pre-treated with 1 μM losmapimod for 1 h before stimulation with 10 ng/mL IL-1β and/or 20 ng/mL TNF-α for 15 min. Cell proliferation was assessed at different time points (3, 5, 7, and 9 days) using AlamarBlue Cell Viability Reagent (Invitrogen) per manufacturer’s instructions. Following 4 h incubation, the plate was read with the SpectraMax iD3 Fluorescence Microplate Reader (Molecular Devices) using 555/595 nm (excitation/emission) filter settings. Trypan Blue solution was used for the determination of cell viability.

### Surface Plasmon Resonance

Surface plasmon resonance (SPR) ligand interactions assays were performed on a Biacore S200 (Cytiva Life Sciences) at 2 °C using multi-cycle settings. Biotinylated avidin-MAPK14 protein (MRC-Reagents, Dundee) was immobilized onto a Streptavidin surface chip, through injection of 50 μg/mL MAPK14 in dimethyl sulfoxide (DMSO)-free SPR running buffer (20 mM HEPES, 150 mM NaCl, 0.1 mM EGTA, 0.5 mM tris(2-carboxyethyl)phosphine (TCEP), 0.01% Tween-20, pH 7.4) over the active flow cell eliciting final captured response units (RUs) of 7719 RUs. The inhibitor analytes (20 mM HEPES, 150 mM NaCl, 0.1 mM EGTA, 0.5 mM TCEP, 0.01% Tween-20, pH 7.4, 1% DMSO) were then injected over both control and active surfaces for 90 s at 30 μL/min before being allowed to dissociate for 600 s over 10 concentration series to record dose responses: 0.05–333.33 nM. A solvent correction was applied to the data collection, and an 8-point DMSO solvent correction was applied. Responses were analyzed using Biacore Evaluation Software (Cytiva Life Sciences) using affinity fit to determine the *K*_d_. Data are representative of three technical replicates.

### Western Blot

Ten micrograms of protein from each sample was mixed with 5× Laemmli buffer with 5% β-mercaptoethanol, heated for 5 min at 100 °C, and separated on 10% sodium dodecyl sulfate (SDS) polyacrylamide gel electrophoresis gels. Following electrophoresis, the proteins were transferred to Immobilon-P transfer membranes (Sigma-Aldrich) using the Trans-Blot TurboTM Transfer System (Bio-Rad Laboratories). Membranes were visualized with Ponceau Red (FlukaTM Analytical). Membranes were probed with the following antibodies purchased from Cell Signaling Technology: MAPKAPK3 (#7421), MAPK14 (#9218), Thr180/Tyr182-P-p38 MAPK (#4511), and anti-rabbit IgG-HRP (#7074). GAPDH (sc-47,724) was purchased from Santa Cruz. The Amersham Imager 600 digital imaging system (GE Healthcare) was used for image acquisition and Quantity One v4.6.3 (Bio-Rad Laboratories) for band densitometry. All western blots are presented as cropped images, with full scans of blots provided in Figure S4.

### Cellular Thermal Shift Assay

Intact-cell TPP experiments were performed on THP-1 cells as described previously, with some slight alterations.^15^ Briefly, 30 million THP-1 cells were treated with 1 μM losmapimod or vehicle (0.01% DMSO) in complete media at 37 °C for 1 h. Cells were collected and washed twice with PBS containing 1 μM losmapimod or vehicle. Washed cells were suspended in PBS supplemented with treatment or vehicle plus 0.4% NP-40, cOmplete Protease Inhibitor Cocktail (Sigma-Aldrich) and phosphatase inhibitor cocktail (1.2 mM sodium molybdate, 1 mM sodium orthovanadate, 4 mM sodium tartrate dihydrate, and 5 mM glycerophosphate) and then separated into eight fractions for thermal profiling. Fractions were heated at 40, 44, 48, 52, 56, 60, 64, and 68 °C for 3 min, incubated for 3 min at room temperature, and snap-frozen at −80 °C. Samples were lysed with four freeze–thaw cycles using dry ice and a thermo block at 35 °C. Cell lysates were centrifuged at 100,000 × *g* for 20 min at 4 °C to separate protein aggregates from soluble proteins. Supernatants were collected, and protein concentrations were determined using Pierce BCA Protein Assay Kit (Thermo Fisher Scientific) for western blot and MS analysis.

### Sample Preparation for MS

#### Reduction, Alkylation, and Digestion of Soluble Fractions for Quantitative Proteomic Analysis

Volumes equivalent to 20 μg of protein per sample were made equal to a final concentration of 5% SDS in 50 mM triethylamonium bicarbonate (TEAB) and 5 mM TCEP (Pierce), incubated for 30 min at 47 °C, and then alkylated with 10 mM iodoacetamide for 30 min at room temperature in the dark. Protein digestion was performed using the suspension trapping sample preparation method according to the manufacturer’s guidelines (ProtiFi, USA). Proteins were digested with trypsin (Worthington-Biochem) in 50 mM TEAB pH 8.0 at an enzyme-to-protein ratio of 1:10 (w/w) for 2 h at 47 °C. Peptides were eluted by three successive washes of 40 μL of 50 mM TEAB pH 8.0, 40 μL of 0.2% formic acid (FA) in water, and 35 μL of 0.2% FA in 50% acetonitrile. The resulting eluates were vortexed and divided into two equal aliquots for LFQ-DIA analysis or TMT preparation and dried before storage at −80 °C.

#### TMTpro-16plex Peptide Labeling and Offline High-Performance Liquid Chromatography Fractionation

Isobaric labeling of peptides was performed using TMTpro 16plex Label Reagent Set (Fisher Scientific, UK) according to the manufacturer’s recommended protocol (lot number: WC320807). After confirming labeling efficiency of >97% by short 1 h liquid chromatography (LC)–MS runs, labeled peptides from each temperature point were combined to a single sample per biological replicate and desalted with C18 Macro Spin Columns (Harvard Apparatus, USA). The pooled sample was subject to fractionation using basic-pH reversed-phase LC on a Gemini C18 column (250 mm × 3 mm, 3 μm, 110 Å; Phenomenex) on a Dionex Ultimate 3000 off-line LC system, generating 12 fractions which were dried. All solvents used were high-performance LC grade (Fisher Scientific). Mobile phase A consisted of 20 mM ammonium formate pH 8.0 and mobile phase B of 100% acetonitrile (MeCN). Peptides were separated over a 49 min linear gradient of 1–49% B at 250 nL/min. Peptide elution was monitored by UV detection at 214 nm. Each of the 12 fractions was acidified to a final concentration of 1% trifluoroacetic acid (TFA) and dried using a speed-vac.

#### Sample Preparation of THP-1 Soluble Fraction Pool for Library Generation

To generate DDA data for project-specific libraries, 60 μg of pooled soluble-fraction digest was subject to fractionation as described above for the TMTpro labeled peptides, generating a total of 12 fractions.

### Liquid Chromatography

All samples (label-free for DIA analysis, or fractioned pools for TMT experiment or DDA library generation) were solubilized in 2% MeCN with 0.1% TFA to 0.2 μg/μL concentration before being injected in volumes equivalent to 1 μg on an UltiMate 3000 RSLC nano System (Thermo Fisher Scientific). Peptides were trapped for 5 min in A (0.1% FA in water) at a flow rate of 10 μL/min on a PepMap 100 C18 LC trap column (300 μm ID × 5 mm, 5 μm, 100 Å) and then separated using an EASY-Spray analytical column (50 cm × 75 μm ID, PepMap C18, 2 μm, 100 Å) (Thermo Fisher Scientific) flowing at 250 nL/min. The column oven temperature was set at 45 °C. All peptides were separated using an identical linear gradient of 3–35% B (80% MeCN containing 0.1% FA) over 120 min.

### Mass Spectrometry

All data were acquired on an Orbitrap Fusion Lumos Tribrid mass spectrometer (Thermo Fisher) in positive ion mode.

#### TMT-Labeled Samples

Data were acquired in DDA mode with positive ion mode. Full MS spectra (*m/z* 375–1500) were acquired at 120,000 resolution, automated gain control (AGC) target 4 × 10^5^, and a maximum injection time of 50 ms. The most intense precursor ions were isolated with a quadrupole mass filter width of 0.7, and higher-energy collision-induced dissociation (HCD) fragmentation was performed in one-step collision energy of 30% and 0.25 activation *Q*. Detection of MS^2^ fragments was acquired in the linear ion trap in rapid scan mode with an AGC target 1 × 10^4^ and a maximum injection time of 50 ms. An electrospray voltage of 2.0 kV and capillary temperature of 275 °C, with no sheath and auxiliary gas flow, was used. Dynamic exclusion of a previously acquired precursor was enabled for 60 s with a tolerance of ±10 ppm. Quantitation of TMT-tagged peptides was performed using FTMS3 acquisition in the Orbitrap mass analyzer operated at resolution of 60,000, with a standard AGC target and maximum injection time of 118 ms. HCD fragmentation of MS^2^ fragments was carried out in one-step collision energy of 55% to ensure maximal TMT reporter ion yield and enable SPS to include 10 MS^2^ fragment ions in the FTMS^3^ scan.

#### Label-Free Samples

Full scan spectra (*m/z* 390–1010) were acquired in centroid mode at an Orbitrap resolution of 60,000, an AGC target set to standard, a maximum injection time of 55 ms, RF lens at 30%, and expected peak width of 20 s. Subsequently, an 8 *m/z* staggered window scheme^24^ was used to collect DIA scans, utilizing 75 windows, with a 4 Da window overlap. HCD collision was set to 33%, loop count of 75, Orbitrap resolution of 15,000, AGC target of 100%, and a maximum injection time of 23 ms.

#### DDA Library Generation

Data were acquired in DDA with positive ion mode. Full MS spectra (*m/z* 350–1000) were acquired at 120,000 Orbitrap resolution, using standard AGC and a maximum injection time of 50 ms. The most intense precursor ions were isolated with a quadrupole mass filter width of 1.6 *m/z,* and HCD fragmentation was performed in one-step collision energy of 30% and 0.25 activation *Q*. Detection of MS^2^ fragments was acquired in the linear ion trap in rapid scan mode with a standard AGC target and a maximum injection time set to auto. Dynamic exclusion of a previously acquired precursor was enabled for 38 s with a tolerance of ±10 ppm.

### Raw Data Processing

#### TMT-DDA Peptide Identification

Raw TMT-DDA files were loaded into MaxQuant (v1.6.10.43)^38^ or FragPipe (v19.1) with MSFragger (v3.7),^36^ Philosopher (v4.8.1),^39^ and IonQuant (v1.7.17)^40^ and searched against the *Homo sapiens* Uniprot database containing 42,426 entries with isoforms (downloaded February 2021). Specific search parameters included trypsin as the protease for digestion and a maximum of two missed tryptic cleavage sites per peptide; dynamic modifications included oxidation of methionine and N-terminal acetylation; and fixed modifications included carbamidomethylation of cysteine residues. Reporter ion MS^3^ was used for quantification. Protein identifications were filtered to a false discovery rate (FDR) of less than 1%, and features matching a contaminant or reverse peptide, only identified by site, or which contained less than two unique peptides were removed. Reporter ion intensity-corrected columns from the MaxQuant dataset were used for downstream TPP data analysis.

#### LFQ-DIA Library Generation

Spectronaut Pulsar (v16.1, Biognosys, Switzerland) was used to construct spectral libraries. DDA raw files were searched with Pulsar to generate a search archive (DDA library). DIA files were subsequently searched in combination with the DDA search archive to produce a hybrid library.

#### LFQ-DIA Peptide Identification

Raw LFQ-DIA files were processed with Spectronaut (v16.1, Biognosys, Switzerland), and either they were analyzed library-free using default setting with DirectDIA or a library-based search was performed using the previously constructed DDA library or hybrid library. Search parameters were identical to those previously specified in MaxQuant search, except MS^2^ was used for quantification. Data filtering was set to *Q*-value and normalization set to automatic. All datasets were filtered in Spectronaut to remove single hits, decoys, and proteins with less than two unique peptides and an FDR of less than 1%. The same DIA files were analyzed library-free using DIA-NN v1.8.1,^36^ or in FragPipe (v19.1) with MSFragger (v3.7),^36^ Philosopher (v4.8.1),^39^ and IonQuant (v1.7.17)^40^ using the DIA_SpecLib_Quant workflow with default parameters and the same FASTA as all other searches. Resulting identifications were filtered using R (v4.0.4) to include only proteins with more than two unique peptides and an FDR of less than 1%.

### Thermal Proteome Profiling Data Analysis

Soluble protein intensity values of each dataset were log2-transformed, normalized to their median abundance, and expressed as a ratio to the lowest temperature sample (40 °C). Each ratio was then normalized to the mean abundance for the identified top 50 temperature-stable proteins in sample 60 °C, as described by Miettinen et al.^41^ The temperature range TPP package was used to perform analysis.^42^ Data were filtered to include only those protein groups suitable for curve fitting (>2 valid fold changes per protein). This fitting was used to determine the melting point (*T*_m_), which is defined as the temperature at which half of the number of proteins was denatured. The melting point differences (Δ*T*_m_) were calculated by subtracting the *T*_m_ values of treated and untreated samples. The sigmoidal melting curves were filtered according to the following criteria: melting curves must reach a relative abundance plateau of <0.3, and the coefficient of determination (*R*^2^) must be >0.8. The significance threshold was set to adjusted *p*-value < 0.05, with a melting point difference of >2 °C and a standard deviation (SD) < 2.

### Data Availability

The mass spectrometry proteomics data have been deposited to the ProteomeXchange Consortium via the PRIDE partner repository with the data set identifier: PXD040173.

### Statistical Analysis

Statistical analyses of data were performed in R (v4.0.4) or GraphPad prism (v9.0.2). Gene Ontology enrichment analysis was performed with g:Profiler with a significance threshold of 0.05.

## Results and Discussion

### Experimental Overview

**Figure 1:**
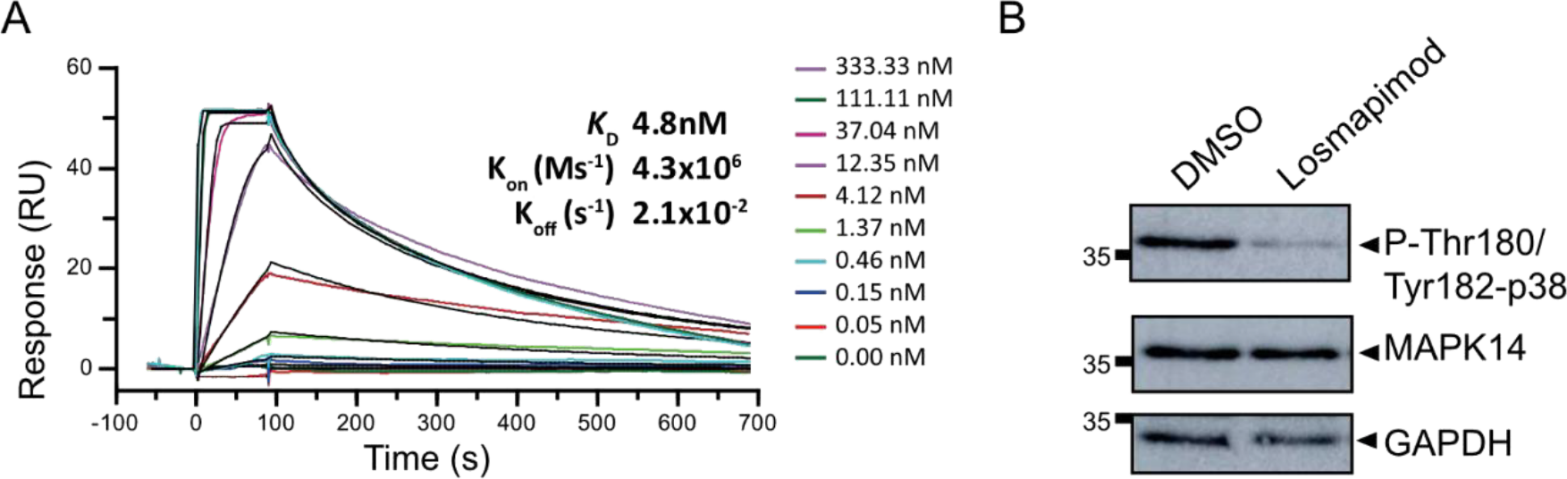
Losmapimod interacts with MAPK14 and reduces p38 MAPK phosphorylation. (A) Assessment of losmapimod binding to MAPK14 using surface plasmon resonance (SPR). Representative multicycle SPR sensograms for losmapimod showing a dose-dependent concentration series against immobilized MAPK14. *K*_D_, equilibrium dissociation rate constant; *K*_on_ (Ms^–1^), on-rate constant or association reaction; *K*_off_ (s^–1^), off-rate constant or dissociation reaction. Experiments were performed in triplicate. (B) Western blot analysis of MAPK14 and p38 MAPK phosphorylation in THP-1 cells with the vehicle control, DMSO, and 1 μM losmapimod for 1 h shows loss of p38 phosphorylation in response to losmapimod. GAPDH serves as a loading control. A representative image of three replicates is shown. Relative mobilities of reference proteins (kDa) are shown on the left of each blot.

The activation of the p38 MAPK pathway plays a critical role in orchestrating a range of cellular stresses, growth, and survival of tumor cells.^43,44^ p38 MAPK is often increased and/or overactivated in AML blasts and represents an important pharmacological target.^45−47^ However, selective p38 MAPK inhibitors have limited efficacy and for a variety of clinical indications none have progressed to Phase III.^48^ Losmapimod (known as GW856553 or GSK-AHAB) is a selective, potent, and orally MAPK14 (p38α MAPK) ATP competitive inhibitor^49^ used in several clinical trials (Table S1). Here, we validated that losmapimod binds directly to MAPK14 by SPR analysis (Figure 1A). In addition, losmapimod had slow dissociation rates (*K*_off_), which have been associated with better selectivity, lower toxicity, and a broader therapeutic window.^50^ Losmapimod was also able to reduce MAPK p38 activation in the human monocytic leukemia cell line, THP-1 (Figure 1B), and reduced the proliferation of AML cells without affecting viability (Figure S1).

To date, losmapimod is the only MAPK14 inhibitor in Phase III clinical trials, as it is safe and well-tolerated in previous human clinical studies. Losmapimod, therefore, represents a valuable therapeutic agent for AML and can provide a positive protein target, MAPK14, for thermal shift analysis and direct method comparison.

**Figure 2:**
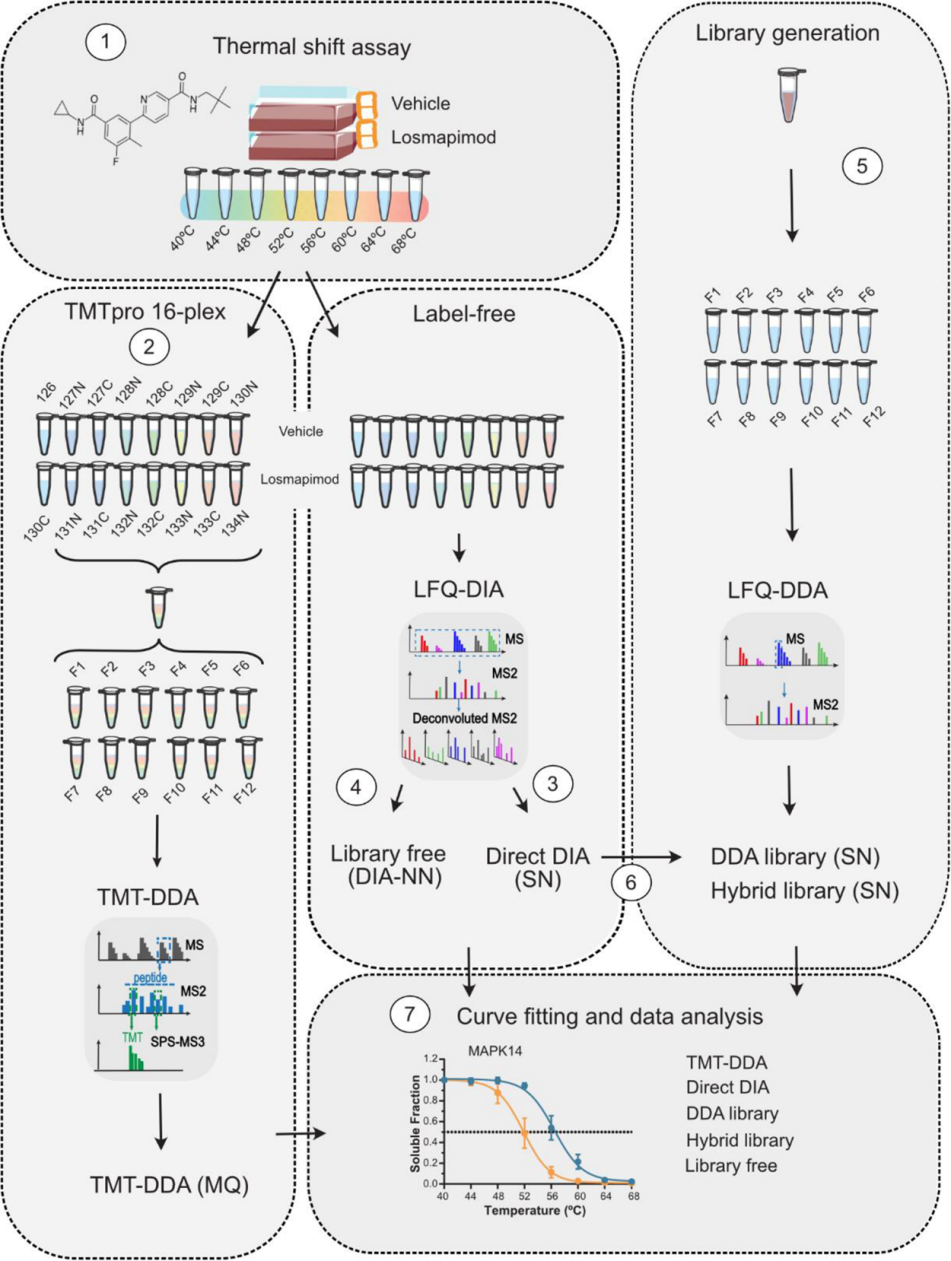
Comparative workflow. (1) To provide a benchmark sample with a positive hit for thermal stabilization, AML cells were treated with 1 μM losmapimod or vehicle for 1 h and then subjected to the thermal shift assay and lysed. Soluble fractions were digested, and each sample split in half for either (2) 16-plex TMT-labeling, fractionation, DDA-MS^3^ quantification, and analysis in MaxQuant (MQ) or for label-free DIA analysis using library-free (3) DirectDIA with Spectronaut (SN) and (4) library-free mode DIA-NN or DDA library-based approaches. For library-generation, small volumes from remaining TPP samples were pooled and digested to provide (5) a project-specific DDA-based library within Spectronaut (SN), and a (6) hybrid library was constructed of both DDA and DIA data. (7) After data processing, thermal proteome profiling data analysis was performed in R and the five workflows compared.

As THP-1 cells have basal activation of MAPK14 (Figure 1B), cells were treated with the compound or vehicle without stimulus in three independent experiments and incubated at eight temperatures between 40 and 68 °C. Denatured, insoluble protein aggregates were then removed by ultracentrifugation, and the protein quantity of each soluble fraction was measured (Figure 2, 1). Equal protein amounts from the soluble-protein fraction were digested and divided in two to compare the performance of all workflows to detect MAPK14 thermal stabilization. TMT-DDA is the traditional approach for melting temperature quantitation in a TPP workflow.^2^ Each temperature for compound and vehicle-treated cells were chemically labeled with an isobaric TMT, using one set of TMTpro 16-plex per replicate. Small aliquots of each sample were pooled and, following clean-up, analyzed by DDA-MS to confirm that the labeling efficiency was >97%. For each replicate, the 16 peptide samples were pooled into a single peptide mixture, cleaned up, and separated into non-consecutively concatenated fractions by basic-pH reversed-phase LC, producing a total of 12 fractions to be analyzed by MS^3^-DDA quantitation (Figure 2, 2).

**Figure 3:**
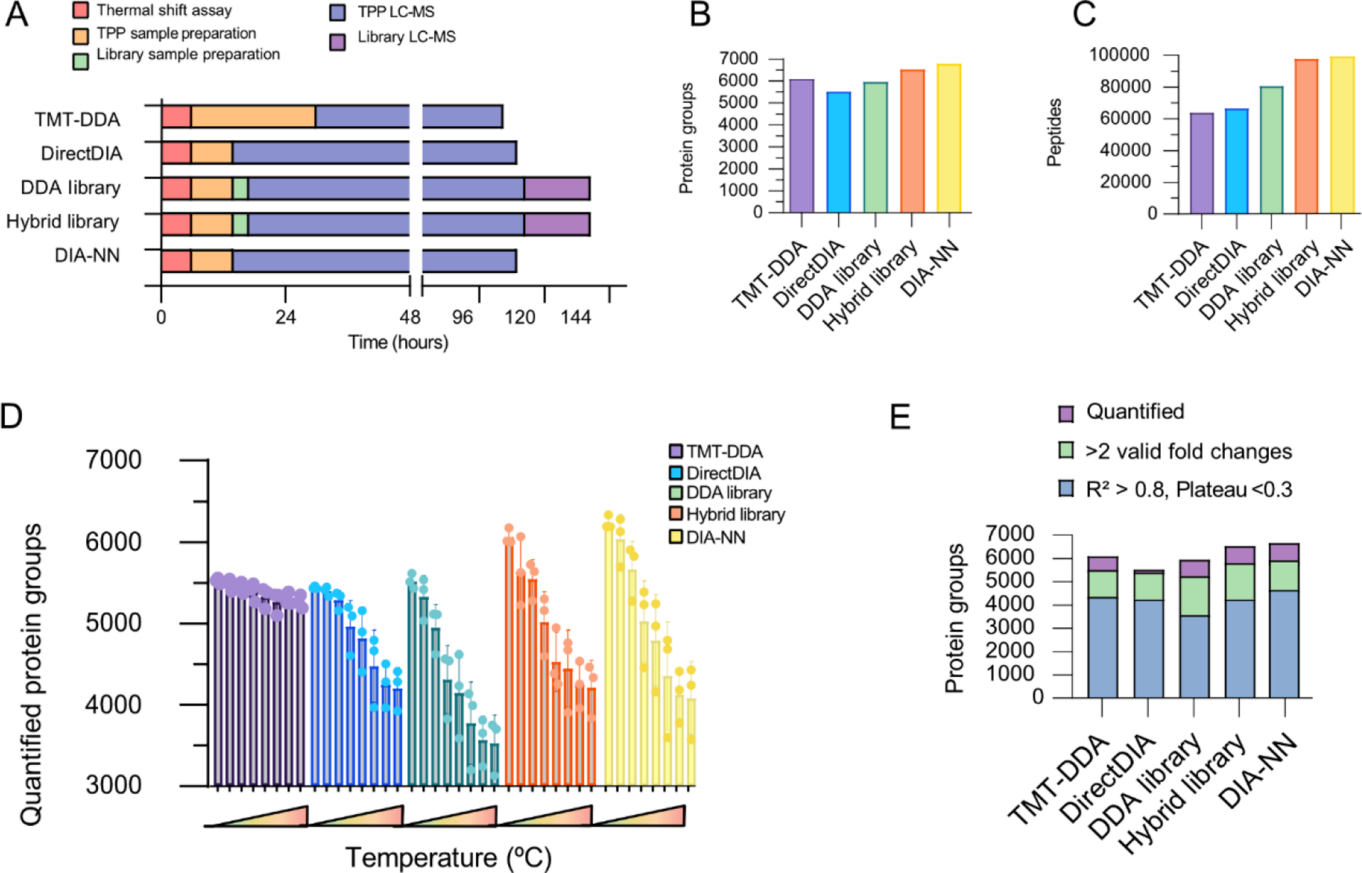
Performance overview of quantitative approaches for thermal proteome profiling. (A) Bar plot showing the time required in hours for each method, comparing thermal shift assay (red), TPP-sample preparation (orange), library-sample preparation (green), instrument time for TPP-sample (blue), and library-sample (purple) analyses. (B) Proteins cumulative across all samples, (C) number of peptides identified, (D) average number of quantified proteins across three biological replicates in each temperature soluble fraction, and (E) average number of quantified proteins (purple), with two valid values for curve fitting (green) and of which sigmoidal curves were fit well (*R*^2^ > 0.8 and plateau <0.3) (blue).

**Figure 4:**
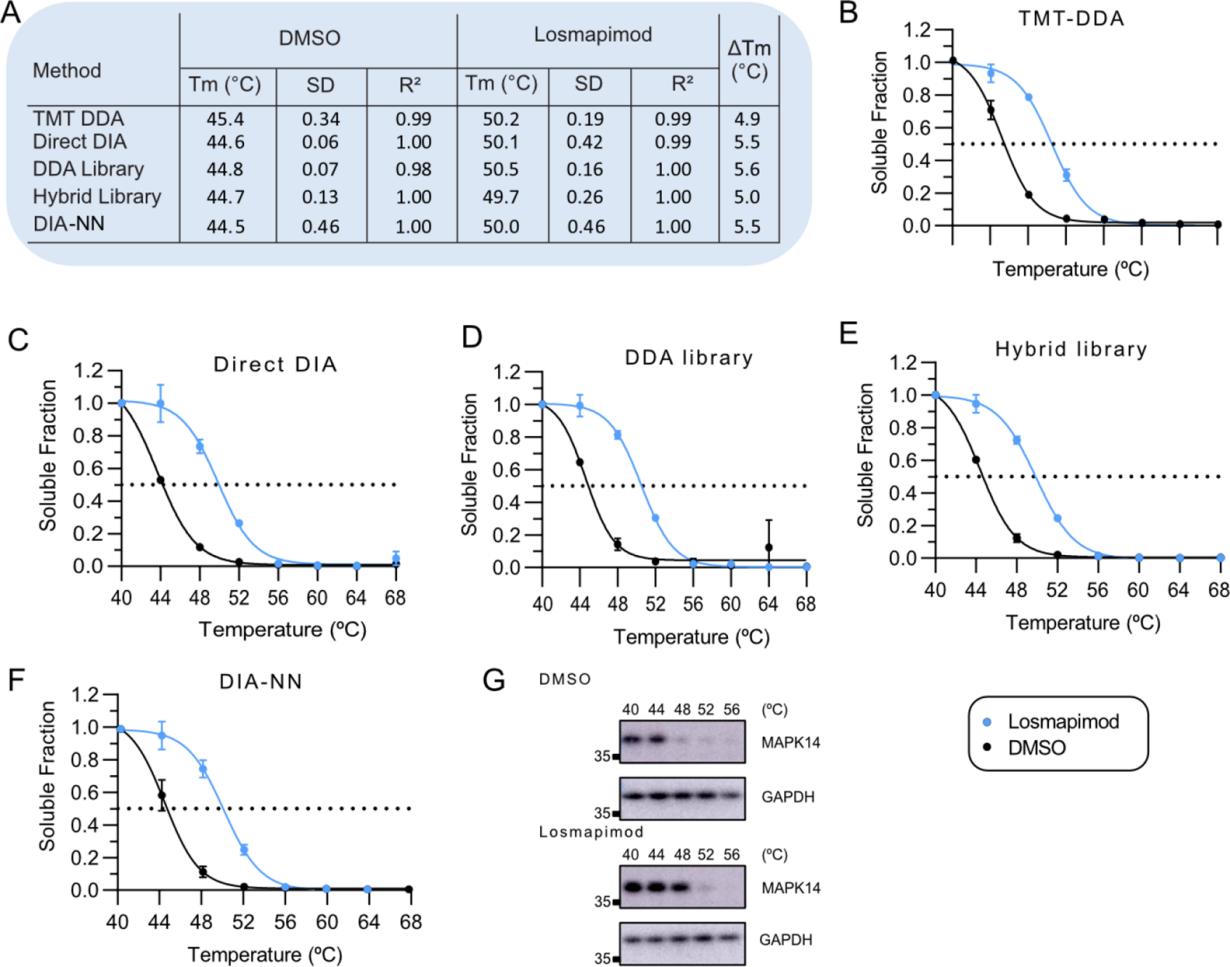
Melting curves for MAPK14. (A) Table with significant shifts in melting temperature (*T*_m_) for MAPK14 quantified by (B) TMT-DDA, (C) Direct DIA, (D) DDA library, (E) hybrid library, and (F) library-free mode DIA-NN after treatment with losmapimod compared to vehicle. Error bars represent the SD of biological replicates. Dashed line indicates melting temperature (*T*_m_), where 50% of the protein is precipitated. (G) Western blot analysis of the MAPK14 at the indicated temperatures in vehicle control (DMSO) and losmapimod treatment. GAPDH served as a loading control. Relative mobilities of reference proteins (masses in kDa) are shown on the left of each blot. SD, standard deviation; *T*_m_, melting temperature; *R*^2^, the coefficient of determination.

The other half of the peptide samples were left unlabeled, analyzed by single-shot DIA, and processed using various different DIA workflows (DirectDIA, DDA library and hybrid library in Spectronaut, and library-free mode in DIA-NN). First, data were searched using DirectDIA for peptide identification (Figure 2, 3). Based on the acquired DIA data, the software generates a pseudo-MS^2^ library by searching data against a sequence database, which is then employed to analyze the original DIA data. This approach provides advantages of reduced instrument time, efforts, and cost by overcoming the need for library generation.^26^ The data were also searched using library-free mode in DIA-NN, which instead generates an in silico library from a protein sequence database (Figure 2, 4).^37^ While their performance have previously been benchmarked and shown unique advantages in large-scale proteomic and phosphoproteomic workflows, they have never been compared for TPP.^27,30,51^

**Figure 5:**
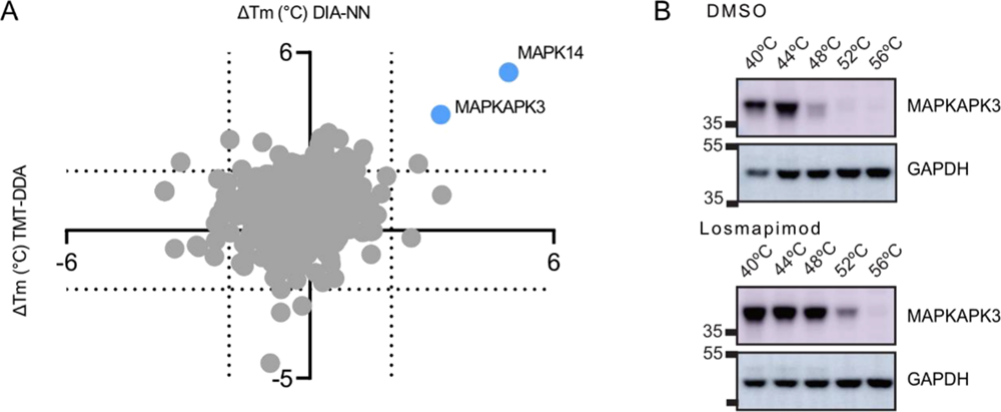
Target deconvolution of losmapimod by TMT-DDA and DIA analysis. (A) Scatter plot of average *T*_m_ shifts calculated from well-fit sigmoidal curves of the three biological replicates following losmapimod treatment. Hits that were identified by both TMT-DDA and DIA-NN, which passed significance criteria are shown in blue. Proteins that did not pass significance criteria are shown in gray. (B) Western blot analysis of the MAPKAPK3 at the indicated temperatures in vehicle control (DMSO) and losmapimod treatment. GAPDH served as a loading control. Relative mobilities of reference proteins (masses in kDa) are shown on the left of each blot.

**Figure 6:**
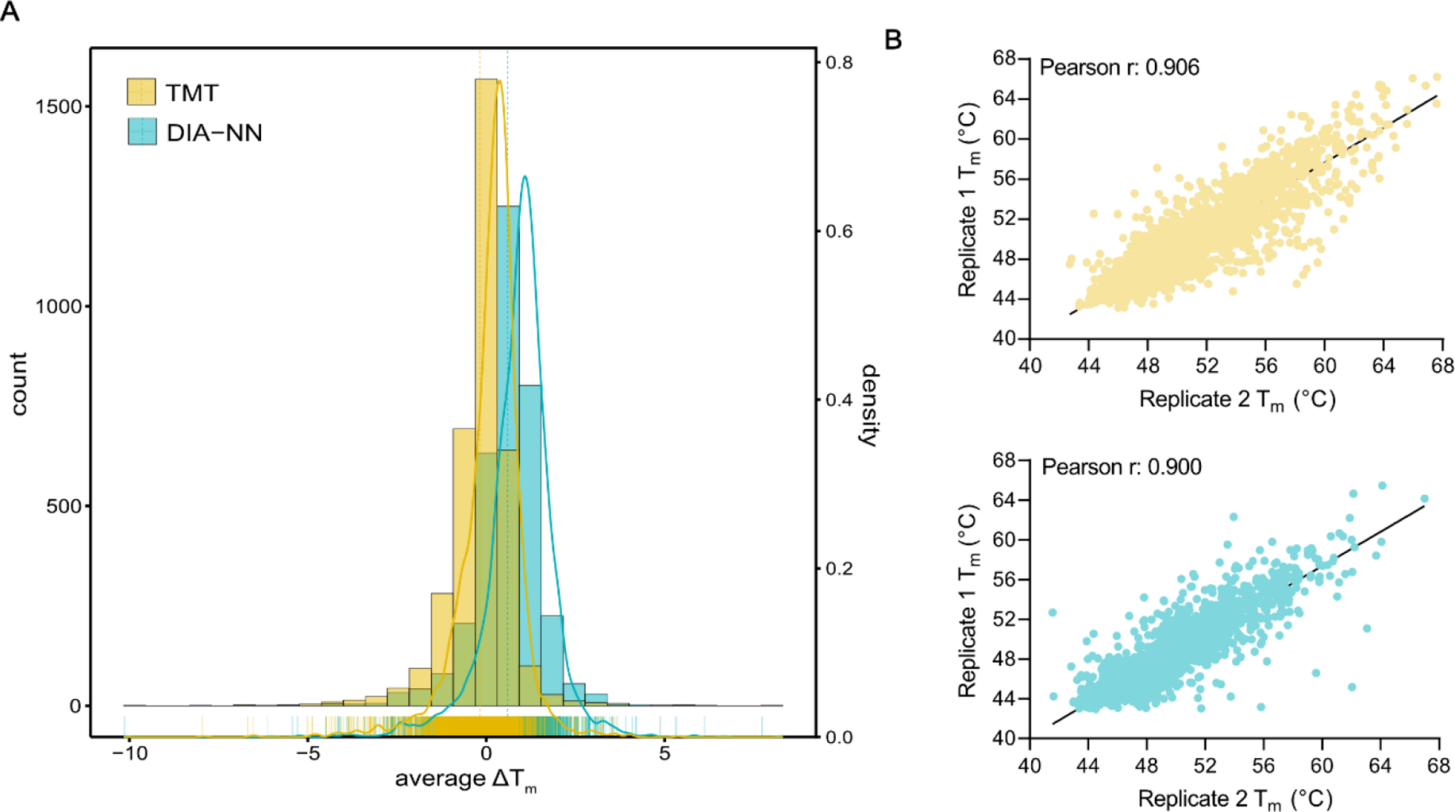
Comparison of measured melting temperatures by TMT-DDA and LFQ-DIA (DIA-NN). (A) Frequency of measured *T*_m_ shifts (°C) (average across three replicates) from all proteins with well-fit sigmoidal curves obtained using TMT-DDA (yellow) and LFQ-DIA (DIA-NN) (blue). (B) Comparison of reproducibility of *T*_m_ measures between replicates 1 and 2 using TMT-DDA (top; yellow) and LFQ-DIA (DIA-NN) (bottom; blue).

Nevertheless, library-based DIA approaches are still largely implemented to improve depth of proteomic coverage in expression studies,^32^ but have also never been explored for thermal shift quantitation. Therefore, the remaining volumes of each soluble fraction were pooled and digested to provide a project-specific reference sample for library generation. Spectral libraries, generated experimentally from peptide fractionation followed by DDA analysis, remain most common in DIA peptide quantification (DDA library).^24,27^ We fractionated the reference sample digest by basic-pH reversed-phase LC, producing 12 fractions to be analyzed by MS^2^-DDA quantification. A DDA library was then constructed in Spectronaut, which consisted of 69,153 precursors, 58,907 peptides, and 7092 protein groups (Figure 2, 5). While DDA library workflows can provide deeper proteome coverage, matrix effects can influence retention time differences between the fractionated, and consequently less complex, DDA samples compared with quantitative single-shot DIA samples. MS^2^ spectra might also differ, as fractionated DDA library spectra will not contain any co-fragmentation interferences that would occur in the original single-shot DIA data.^24^ Nonetheless, we generated a hybrid library, by performing DirectDIA with the addition of DDA data, which achieved greater proteome coverage, as the library consisted of 124,908 precursors, 99,121 peptides, and 8285 protein groups (Figure 2, 6). All samples were run on identical analytical gradients and resulting thermal proteome profiling analysis performed in R (Figure 2, 7).

### Protein Identification

There is a fundamental trade-off between achieving deep proteomic coverage and obtaining suitable throughput in proteomics studies, where chemoproteomics is no exception. The overall time required for each workflow was compared (Figure 3A and Table S2A). In total, the thermal shift assay produced 48 samples (two treatment conditions: losmapimod and vehicle, each with eight temperatures and three biological replicates) that were either analyzed by label-free single-shot DIA-MS or further labeled with TMT.

As shown in Figure 3A, the DIA workflows (including TPP sample preparation, any library-sample preparation, TPP-sample MS time or library acquisition MS time) consumed 110 h (Direct DIA and DIA-NN) and 137 h (DDA library and hybrid library). Cumulatively, TMT quantification required 105; 24 h for sample preparation, to label peptides of each biological replicate with a batch of 16-plex TMT, introducing additional clean-up steps, whereas label-free single-shot DIA required only 8 h of sample preparation. Labeling efficiency checks were performed, and quality was checked before sample preparation could proceed, adding 3 h of instrument time to the TMT experiment. However, given the established performance of TMT labeling protocols in laboratories that regularly employ them, efficiency checks may not always be conducted for every run. The three sets of labeled samples were pooled, cleaned up, and fractionated, which took 5 h, due to pools having larger sample volumes. This totaled 39 samples, which altogether took 105 h of method time. Despite more sample handling time, TMT-DDA was the quickest method overall, with 75 h LC–MS time compared with 96 h LC–MS/MS analysis for the DIA TPP samples. Various factors may influence the decision of which DIA workflow to adopt, such as number of compounds/conditions and availability to instrument time and so would need to be assessed on a project-specific basis. Nevertheless, the capacity for investigating the target landscape of multiple compounds, effects of combination therapies, or resistance mechanisms becomes more feasible with a label-free approach or if within the same biological model, a project-specific library could be repurposed, becoming increasingly cost-effective.

Each approach identified a different number of cumulative protein identifications (Figure 3B; Table S2B). Overall, library-free DIA-NN yielded the greatest number of protein groups (6669 protein groups) and peptide identifications (99,678 peptides), while DirectDIA data provided the lowest (5518 protein groups; 66,612 peptides). TMT-DDA data, searched with MaxQuant (6086 protein groups), identified more protein groups compared to the DDA library analysis of the DIA data, likely reflecting the off-line fractionation, highlighting an advantage of the workflow and corroborating a previous benchmark comparison.^11^ However, the hybrid library (6528 protein groups; 97,815 peptides) outperformed the DDA library (5954 protein groups; 80,630 peptides) as library performance is influenced directly by its quality and coverage, and the hybrid library consisting of both DDA and DIA raw files was the most in-depth (Figure 3C).^48^ Furthermore, to explore the impact of software selection on protein identification, an alternative software, Fragpipe, was used to process TMT-DDA and LFQ-DIA data. The default TMT-16 MS3 and DIA_SpecLib_Quant workflows were used for the respective datasets. When TMT-DDA data were processed using Fragpipe, slightly more protein identifications were obtained compared to MaxQuant (6287 vs 6086 protein groups) (Table S3A). However, Fragpipe did not perform as well as DIA-NN, when processing LFQ-DIA data (5760 vs 6669 protein groups) (Figure S2A; Table S3B). Due to the relatively small differences in protein identifications achieved with Fragpipe compared to other software options, further comparative analysis using Fragpipe was not pursued.

Considerable overlap in identified protein groups was seen between all approaches, although less so between DIA approaches and TMT-DDA (Figure S2B). This finding highlights that DIA-based methods analyze slightly different portions of the proteome to DDA, as on average 67% of protein groups were common between DIA approaches and TMT-DDA, as previously reported.^52^ In DDA mode, the most abundant precursor peptide ions are isolated for acquisition of MS^2^ spectra, whereas the entire *m/z* range is selected in DIA, for an unbiased set of precursors, leading to variations in peptide, and ultimately protein identification. Moreover, peptides acquire a shift in mass and charge because of isobaric labeling, resulting in different peptides being fragmented. A comparison in protein identifications between the two best performing workflows, TMT-DDA with MaxQuant and LFQ-DIA with DIA-NN, was subject to gene ontology (GO) enrichment analysis to explore any bias in proteins being identified by either approach (Figure S2C). The results indicated that most significant GO terms were common between the two approaches, suggesting that there were only marginal differences in the functionality in protein groups identified by the two methods. However, there were also some exclusive GO terms observed (Figure S2D; Table S4). Interestingly, the DIA-NN analyses exhibited an enrichment of proteins associated with the mitochondria or kinase activity. This finding suggests that the LFQ-DIA approach using DIA-NN may have specific advantages for studies of kinase targeting compounds or effects on mitochondrial processes.

### Melting Curve Fitting

Measuring significant shifts in protein thermal stability, indicative of compound-target engagement, relies on the successful construction and reproducible analysis of full protein melting curves. Therefore, the computational analysis of TPP datasets requires unique consideration. Raw abundance values from DDA or DIA analysis are quality-filtered prior to curve fitting to exclude low-confidence identifications by number of peptide-spectrum matches or unique peptide number. As temperature increases, identification profiles become sparser by way of protein aggregation and the complexity of the soluble fractions becomes reduced.^53^ TMT-DDA identified more protein groups in the less complex, higher-temperature samples than all DIA approaches (Figure 3D). However, data are required to have two valid fold changes (and must therefore be detected in the lower reference temperature) for plotting the melting curve. Despite fewer missing values across the high-temperature profiles, several protein groups were not detected in the lower reference temperature for TMT-DDA, resulting in 5497 protein groups suitable for curve fitting. DIA-NN provided more curves suitable for fitting (5961 protein groups) along with the hybrid library analysis of DIA data (5230 protein groups) (Figure 3E, green; and Table S2B).

Resulting sigmoidal curves were filtered for reliable protein melting point calculation prior to interpretation of the statistical and significance comparison (Tables S5–S9). Only curves with a minimum coefficient of determination of <0.8, indicating how well the fold changes fit the melting curve and a plateau (lower horizontal asymptote) of <0.3, were included, as recommended by Franken et al.^2^ TMT-DDA accurately quantified melting temperatures of 4497 protein groups on average, performing comparably to DIA-NN, generating an average of 4458 melting curves (1% less) (Figure 3E, blue). Interestingly, library-free mode with DIA-NN performed better than DirectDIA and hybrid library (both 6% less relative to TMT-DDA). Finally, despite providing more protein identification than DirectDIA, DDA-library based analysis of the DIA dataset performed worst with a 20% reduction in well-fit melting curves compared to DIA-NN and TMT-DDA, highlighting the significance of reliable quantification over number of protein identifications for TPP pipeline output. In fact, both library-free DIA approaches performed either equally or better than library-based DIA methods.

### Performance for Target Deconvolution

Results were filtered to include proteins with a significantly positive or negative fold change in melting temperature, of greater than 2 °C, and a change in melting temperature (*T*_m_) standard deviation of less than two. As expected, the known target of losmapimod, MAPK14, was stabilized following treatment and detected by all workflows (Figure 4A–F). On average, DIA approaches measured MAPK14 *T*_m_, which increased from 44.6 ± 0.1 °C in vehicle controls, consistent with previous reports,^15^ to 50.0 ± 0.3 °C with losmapimod. This was independently validated by western blot (Figure 4G). Notably, the average *T*_m_ for MAPK14 after DMSO treatment measured by TMT-DDA was up to 0.9 °C higher compared with DIA, possibly due to co-isolation of precursors reducing quantification accuracy, despite using the SPS-MS^3^ for acquisition;^11^ that is, an under-measure of thermal shift could be an artifact to ratio compression.^6^ DIA approaches that measured a greater thermal shift suggest that label-free DIA may be more sensitive to subtle changes in thermal stability.

Given the therapeutic potential of losmapimod for the AML, we also explored its off-target landscape while evaluating the performance of all five pipelines. In addition to MAPK14, the downstream phosphorylation and interaction target MAPKAPK3 was significantly stabilized and detected by all methods. As seen in Figure 5, DIA-NN again detected a larger shift in melting temperature (+3.9 ± 0.1 °C) in MAPKAPK3, following losmapimod treatment compared with TMT-DDA (+3.2 ± 0.1 °C). Thermal stabilization in this intact cell-based experiment may be due to inhibitor-induced biological changes in intracellular signaling.^6^ Phosphorylated proteins can display a different melting profile compared to their non-phosphorylated equivalents.^6,54^ To distinguish whether effects were primary (a consequence of direct drug binding) or secondary (a downstream cellular response to treatment), TPP in cell extracts without functioning PTM cellular machinery would need to be performed.^4^ MAPKAPK3 stabilization was independently validated by western blot (Figure 5B).

Furthermore, TMT-DDA detected significant thermal stabilization of myosin light chain kinase (MYLK) (3.6 ± 0.6 °C) following treatment with losmapimod, but the protein was not detected in any DIA datasets (Figure S3A). A recent study reported MYLK to be a potentially new intracellular interaction partner of MAPK14,^55^ which may have been stabilized by association due to MAPK14 target engagement. Stability changes in proteins present in a complex have been identified previously with TPP, for example, kinase complexes containing cyclins were stabilized by the kinase inhibitor staurosporine.^4^ TMT-DDA was also the only approach to detect significant thermal destabilization of RAC-gamma serine/threonine-protein kinase (AKT3) with a shift in melting temperature from 53.6 ± 2.2 to 49.7 ± 2.9 °C, following losmapimod treatment (Figure S3B). Of note, measured melting temperatures for AKT3 had great variability between biological replicates, and if the significance threshold is increased to >0.01, the hit is removed. Although AKT3 was detected in the DIA data, its quantification did not pass the filtering criteria and was not used for statistical comparison, indicating that TMT-DDA is more tolerant to noise. Overall, these results demonstrate the great specificity of losmapimod as a therapeutic agent, while demonstrating the potential of DIA to measure significant changes in thermal stability.

We assessed the range of thermal shifts obtained from LFQ-DIA and TMT-DDA for all proteins with reliable melting curves. Given the use of a highly specific kinase inhibitor, it was anticipated that most thermal shifts would be zero. On average, thermal shifts were marginally higher (0.6 ± 1 °C) with the LFQ-DIA method compared to TMT-DDA (−0.2 ± 1 °C), indicating slightly less quantitative accuracy (Figure 6A). Determination of melting points at higher temperatures exhibited lower precision with LFQ-DIA when compared to TMT-DDA (Figure 6B). It is generally recognized that DIA approaches perform optimally when the samples being compared exhibit a relatively similar protein composition, as accurate quantification in label-free DIA relies on comparable peptide profiles among samples, that is, at lower temperatures, most human proteins do not undergo extensive melting; therefore, samples will not be too dissimilar. In cases where samples exhibit substantial differences in protein composition, such as at higher temperatures when many human proteins will have reached *T*_m_,^56^ accuracy of LFQ-DIA quantification may be compromised. In the context of higher temperature ranges, TMT-DDA offered slightly improved accuracy and reliability which should be considered in experimental design.

## Conclusions

In summary, we evaluated various DIA workflows that have not previously been compared for a TPP pipeline and benchmarked their performance with traditional TMT-DDA. This study highlights the potential of label-free DIA quantitative MS approaches for target deconvolution. There were advantages in using different DIA approaches, such as increased proteomic coverage by a library-based approaches or improved throughput from library-free analysis; in comparison to TMT-DDA, they required less sample preparation time, but more instrument run-time, as expected with single-shot analyses. Ultimately, all methods compared in this study were unanimously able to identify MAPK14 as the primary target of the compound of interest, as well as detect MAPKAPK3 stabilization. Smaller thermal shifts were observed by TMT-DDA compared to DIA, potentially a consequence of ion-interference or ratio compression. TMT-DDA identified two additional protein hits; one of which was thermally stabilized but not detected by DIA, which is a caveat of comparing different acquisition approaches. The second additional hit suffered from increased variability between replicates and was therefore filtered out in the DIA dataset, suggesting that TMT-DDA data may be more tolerant to noise. Comparison of Spectronaut and DIA-NN software for library-free DIA for TPP revealed differential performance, as DIA-NN provided more well-fit melting curves, although software for the processing of DIA data is continually being developed and results may be different in alternative software versions. Nevertheless, given its superior performance and open-access, DIA acquisition using library-free mode DIA-NN is a practical and cost-effective method for thermal proteome profiling.

## Supporting Information Available

Losmapimod inhibiting p38α and proliferation but not viability in AML cells, comparative analysis of protein identification approaches, quantification of thermostability of MYLK3 and AKT, unprocessed images of all Western blots, summary of clinical trials of losmapimod, systematic comparison of the performance of five quantitative workflows for thermal proteome profiling, MS-Fragger performance for protein identification, gene ontology enrichment analysis for proteins exclusively identified by either TMT-DDA (MaxQuant) or LFQ-DIA (DIA-NN), TMT-DDA TPP analysis, DirectDIA TPP analysis, DDA library TPP analysis, hybrid library TPP analysis, and DIA-NN TPP analysis (ZIP)

## Abbreviations

AGC: automated gain control
AKT3: RAC-gamma serine/threonine-protein kinase
AML: acute myeloid leukemia
DDA: data dependent acquisition
DIA: data independent acquisition
FA: formic acid
FDR: false discovery rate
FDR: false discovery rate
FTMS: Fourier transform mass spectrometry
GAPDH: glyceraldehyde 3-phosphate dehydrogenase
HCD: higher-energy collision-induced dissociation
IL: interleukin
LC–MS: liquid chromatography–mass spectrometry
LFQ: label-free quantification
m/z: mass-to-charge ratio
MAPK: mitogen-activated kinase
MAPKAPK: MAPK-activated protein kinase
MeCN: acetonitrile
MS: mass spectrometry
MS2: tandem mass spectrometry or MS/MS
MYLK: myosin light chain kinase
NP-40: Tergitol-type NP-40 and nonyl phenoxypolyethoxylethanol
PBS: phosphate buffered saline
RUs: response units
SD: standard deviation
SDS: sodium dodecyl sulfate
SDS-PAGE: sodium dodecyl sulfate-polyacrylamide gel electrophoresis
SPR: surface plasmon resonance
SPS: synchronous precursor selection
TCEP: tris(2-carboxyethyl)phosphine
TEAB: triethylamonium bicarbonate
TFA: trifluoroacetic acid
TKIs: tyrosine kinase inhibitors
*T*_m_: melting temperature
TMT: tandem mass tags
TNF-α: tumor necrosis factor alpha
TPP: thermal proteome profiling
UV: ultraviolet
