# Supplementary material for: Comparison of Quantitative Mass Spectrometric Methods for Drug Target Identification by Thermal Proteome Profiling
Source: J Proteome Res. 2023 Jul 13;22(8):2629–40. doi: 10.1021/acs.jproteome.3c00111 (PMC10407934; doi:10.1021/acs.jproteome.3c00111)
Supplement: Supplementary file 1 — pr3c00111_si_001.zip [file pr3c00111_si_001.zip › Supporting Information/Supporting Information.docx]

**Supporting Information for**

**A Comparison of Quantitative Mass Spectrometric Methods for Drug Target Identification by Thermal Proteome Profiling**

Amy L. George^1^, Frances R. Sidgwick^1^, Jessica E. Watt^2^, Mathew P. Martin^2^, Matthias Trost^1*^_,_ José Luis Marín-Rubio^1*^_,_ Maria Emilia Dueñas^1*^.

^1^Laboratory for Biological Mass Spectrometry, Biosciences Institute, Newcastle University, Newcastle-upon-Tyne, NE2 4HH, UK

^2^Newcastle Cancer Centre, Northern Institute for Cancer Research, Medical School, Newcastle University, Paul O’Gorman Building, Framlington Place, Newcastle upon Tyne NE2 4HH, UK.

* Corresponding authors (matthias.trost@ncl.ac.uk; jose.marin-rubio@newcastle.ac.uk; maria.duenas@newcastle.ac.uk)

Table of Contents

[Supplementary Figures 4](#_Toc136542039)

[**Figure S1**. Losmapimod inhibits p38α and proliferation but not viability in AML cells. 4](#_Toc136542040)

[**Figure S2**. Comparative Analysis of Protein Identification Approaches 5](#_Toc136542041)

[**Figure S3.** Quantification of thermostability of **A)** MYLK3 and **B)** AKT, detected by TMT-DDA. Error bars represent the SD of three biological replicates. 5](#_Toc136542043)

[**Figure S4.** Unprocessed images of all Western blots as indicated. Molecular size markers in kDa. 6](#_Toc136542044)

[Supplementary Tables 7](#_Toc136542045)

[**Table S1**. Summary of clinical trials of losmapimod. 7](#_Toc136542046)

[**Table S2**. A systematic comparison of the performance of five quantitative workflows for thermal proteome profiling.](#_Toc136542047)

[**Table S3. MS-Fragger performance for protein identification. A)** TMT-DDA Protein Identification using Fragpipe: TMT-16 MS3 **B)** LFQ-DIA Protein Identification using Fragpipe: DIA_SpecLib_Quant 7](#_Toc136542048)

[**Table S4.** Gene Ontology Enricment Analysis for proteins exclusivley identified by either TMT-DDA (MaxQuant) or LFQ-DIA (DIA-NN). 7](#_Toc136542049)

[**Table S5.** TMT-DDA TPP analysis. 7](#_Toc136542050)

[**Table S6.** DirectDIA TPP analysis. 7](#_Toc136542051)

[**Table S7.** DDA library TPP analysis. 7](#_Toc136542052)

[**Table S8.** Hybrid library TPP analysis. 7](#_Toc136542053)

[**Table S9.** DIA-NN TPP analysis. 7](#_Toc136542054)

#
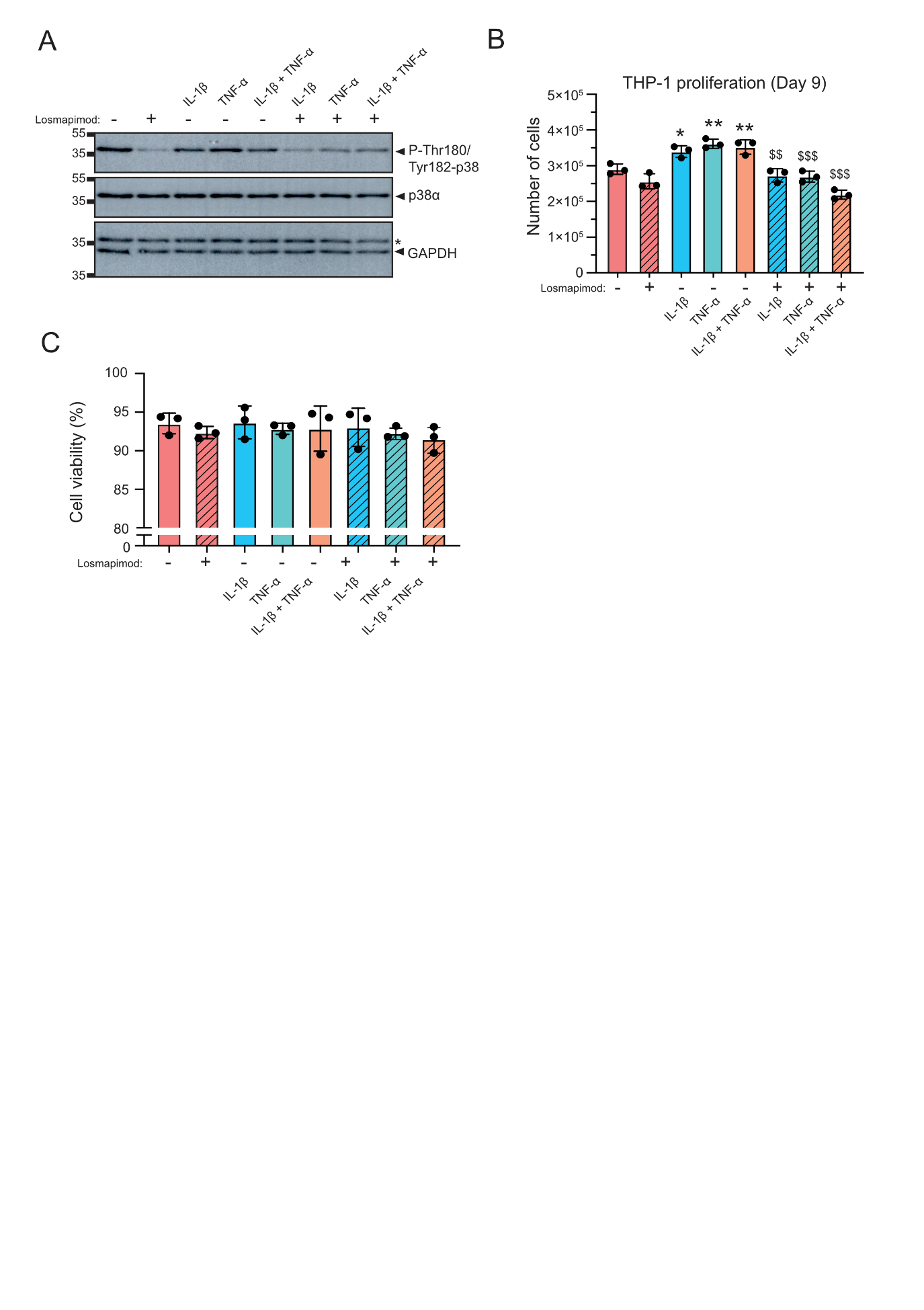
Supplementary Figures

## **Figure S1**. Losmapimod inhibits p38α and proliferation but not viability in AML cells.

**A)** Western blotting of the p38-MAPK phosphorylation in THP-1 cells pretreated with 1 µM losmapimod for 1 h before stimulation with 10 ng/mL IL-1β and/or 20 ng/mL TNF-α for 15 min. GAPDH serves as a loading control. A representative image with three biological replicates is shown. Relative mobilities of reference proteins (masses in kDa) are shown on the left the blot. **B)** Proliferation assay at day 9 in THP-1 cells with 1 µM losmapimod for 1 h before stimulation with 10 ng/mL IL-1β and/or 20 ng/mL TNF-α for 9 days. One-way ANOVA tests were performed (*, p<0.05; **, p<0.01; samples compared to untreated-sample without losmapimod; $$, p<0.01; $$$, p<0.001 samples compared to treated-samples without losmapimod). Error bars represent the standard deviation of three biological replicates. **C)** Cell viability assay at day 9 in THP-1 cells treated with 1 µM losmapimod for 1 h before stimulation with 10 ng/mL IL-1β and/or 20 ng/mL TNF-α. One-way ANOVA tests were performed. Error bars represent the standard deviation of three biological replicates.

**
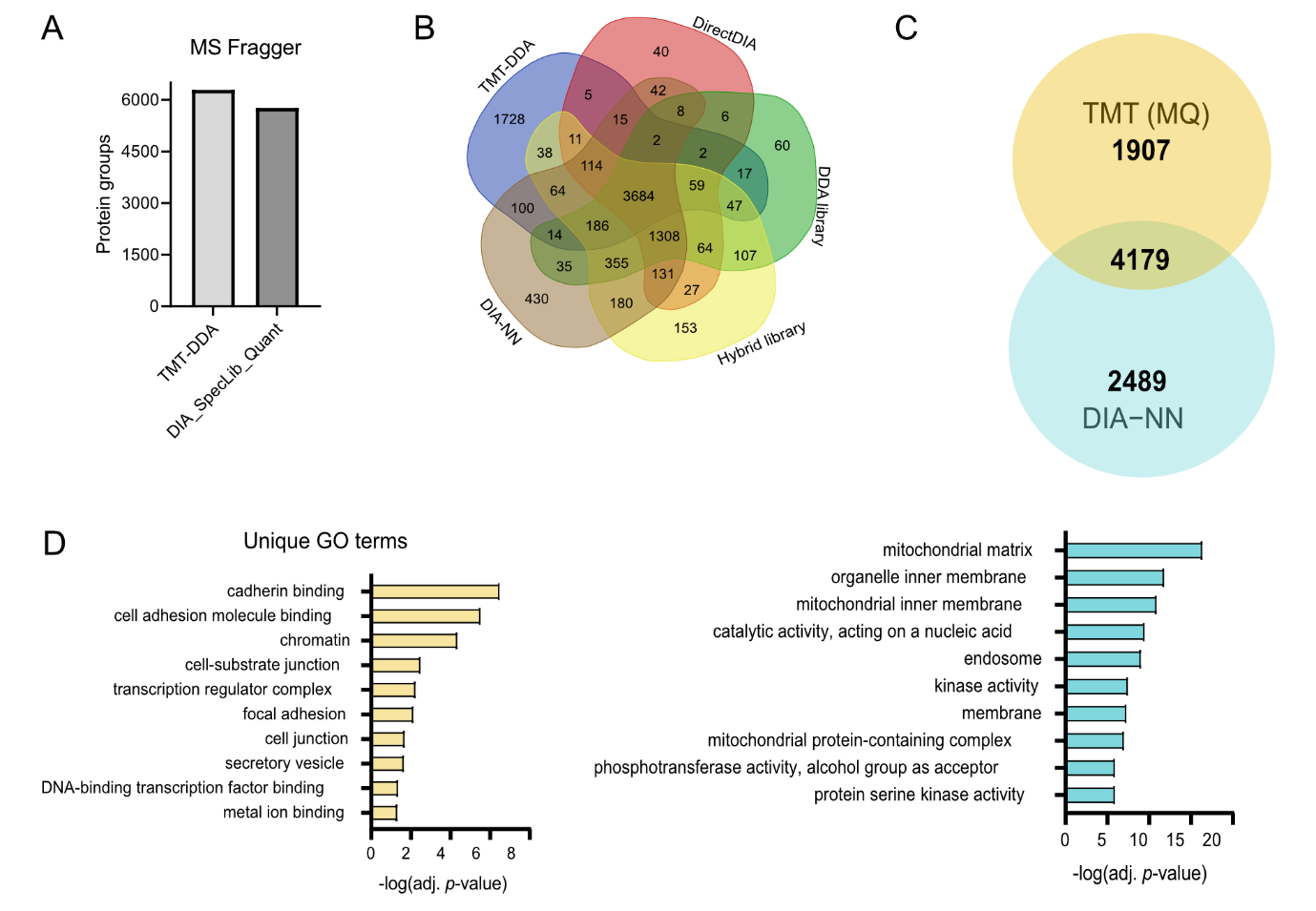
**

## **Figure S2**. Comparative analysis of protein identification approaches.

## **A)** The number of protein groups identified using Fragpipe TMT-MS^3^ or DIA_SpecLib_Quant workflow for processing TMT-DDA and LFQ-DIA data, respectively. **B)** A Venn diagram comparing the cumulative protein groups identified across different approaches. **C)** A Venn diagram comparing all protein groups identified between TMT-DDA (MaxQuant) and LFQ-DIA (DIA-NN). **D)** Gene Ontology enrichment analysis results, showing exclusive biological processes and cellular compartment terms enriched in proteins identified by either the TMT-DDA (using MaxQuant; left, yellow) or LFQ-DIA (using DIA-NN, right, blue) method.


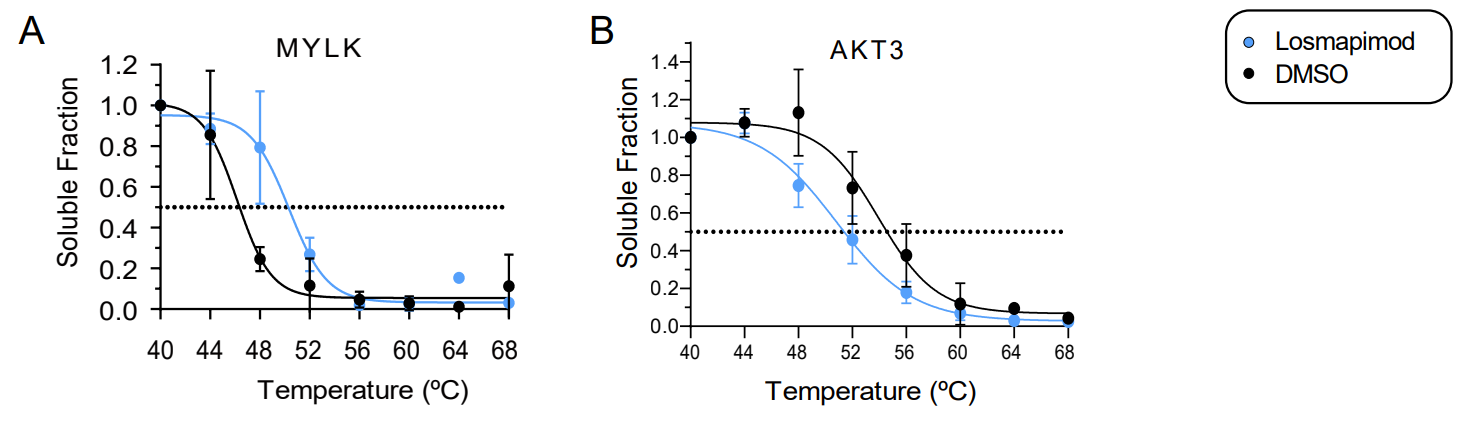


## **Figure S3.** Quantification of thermostability of **A)** MYLK3 and **B)** AKT, detected by TMT-DDA. Error bars represent the SD of three biological replicates.


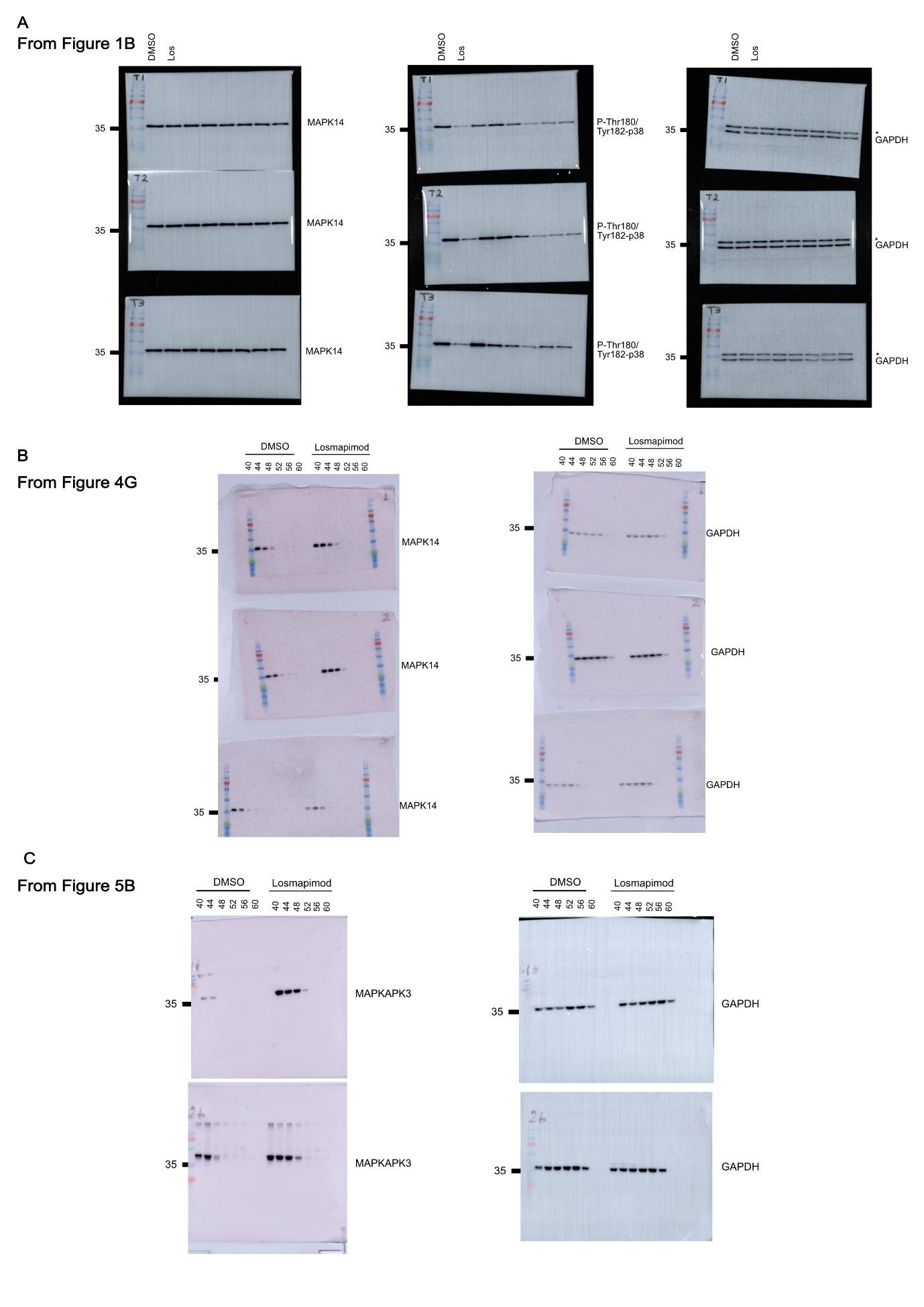
**Figure S4**. Unprocessed images of all Western blots as indicated. Molecular size markers in kDa.

# Supplementary Tables

## **Table S1**. Summary of clinical trials of losmapimod. A clinicaltrials.gov search on September 22, 2022, for losmapimod (keywords: losmapimod, GW856553, GW856553X, SB856553 or GSK-AHAB).

## **Table S2**. A systematic comparison of the performance of five quantitative workflows for thermal proteome profiling. **A)** The time required for each workflow, categorised by hours required for sample preparation, TPP analyses and any library generation. **B)** A breakdown of the total number of protein groups identified by each workflow, and resulting melting curves.

## **Table S3. MS-Fragger performance for protein identification. A)** TMT-DDA Protein Identification using Fragpipe: TMT-16 MS3 **B)** LFQ-DIA Protein Identification using Fragpipe: DIA_SpecLib_Quant

## **Table S4.** Gene Ontology Enricment Analysis for proteins exclusivley identified by either TMT-DDA (MaxQuant) or LFQ-DIA (DIA-NN).

## **Table S5.** TMT-DDA TPP analysis.

## **Table S6.** DirectDIA TPP analysis.

## **Table S7.** DDA library TPP analysis.

## **Table S8.** Hybrid library TPP analysis.

## **Table S9.** DIA-NN TPP analysis.
